# The Construction of Online Course Learning Model of Piano Education from the Perspective of Deep Learning

**DOI:** 10.1155/2022/4378883

**Published:** 2022-02-12

**Authors:** Nansong Huang, Xiangxiang Ding

**Affiliations:** ^1^Xi'an Conservatory of Music, Xi'an 710061, Shaanxi, China; ^2^Xi'an Institute of Physical Education, Art College of Xi'an Institute of Physical Education, Xi'an 710068, Shaanxi, China

## Abstract

This exploration aims at solving multiple teaching problems in piano online education course. On the premise of collaborative filtering, the K-means clustering algorithm is employed to apply the time data to the neural collaborative filtering algorithm, and the Improved Neu Matrix Factorization (Improved Neu MF) algorithm model is implemented. After the experiment, the relevant evaluation indexes are selected and the simulation test is operated on the relevant dataset. The test results show that root mean square error (RMSE) reaches 1.251 and mean absolute error (MAE) is 0.625. Indexes are adopted to evaluate the order of the model. The results suggest that the designed algorithm is better than the comparison algorithm, proving that the optimized model has better performance and can be used to construct an online course model. Based on deep learning, using the designed algorithm to build the online learning model of piano education can provide better, dynamic, and personalized online course recommendations for piano education. In this way, it can improve students' learning efficiency, promote the online learning development of piano education, and have vital practical significance for disseminating art and culture.

## 1. Introduction

In the information age, with the continuous economic development [1], the demand for art and culture at all levels is gradually showing a straight-line upward trend after people's basic physiological needs are met. Piano, an important member of music, is crucial for cultivating people's sentiment. Piano education [2] has two modes, social music education and school music education, whose joint action promotes the development of piano education. Online education provides it with a new education model in the network age. The network brings convenience and quickness and multiple additional problems for piano education. For example, teachers themselves cannot intuitively observe learners' learning status. It is difficult for them to collect learners' learning degree information after class, so they cannot bring learners more curriculum information according to their needs. In fact, teachers accumulate massive valuable data when using network teaching [3]. The recommendation system provides a good solution for using these data to feedback to the education itself.

Integrating the advantages of different recommendation algorithms is the idea of establishing the recommendation system, so the performance of recommendation algorithm reflects whether the performance of recommendation system is excellent or not. The earliest recommendation algorithm aims at bringing personalized movie viewing services to people watching movies and launch a movie recommendation system. Later, some scholars used collaborative algorithms as a crucial technology in the recommendation system. The goods-based collaborative filtering algorithm is introduced based on the former. It gradually has related classifications with the continuous development of recommendation system, such as content, collaborative, filtering, and hybrid intelligent recommendation methods. They develop progressively in teaching courses, providing multiple possibilities for online education and personalized recommendation education [4]. The disadvantage is that the accuracy of the recommendation algorithm will decline for missing data values, and it will also face the problem of data sparsity.

This exploration is to further solve the problem of data sparsity on the premise of collaborative filtering. The advantages of deep learning and traditional collaborative filtering recommendation algorithm are combined to design a neural collaborative filtering algorithm. The linear relationship between users and items is found through the traditional matrix factorization (MF). The nonlinear relationship between them is found through a multilayer neural network to reduce the interference of missing data values and optimize the accuracy. Curriculum resources and learning should be changed accordingly when teaching continues. The K-means clustering algorithm is employed to apply time data to a neural collaborative filtering algorithm to optimize online course resources to the greatest extent [5]. After the experiment, the relevant evaluation indexes are selected to operate the simulation test on the relevant dataset. The test results show that the model has an optimized performance. The model designed by using a neural collaborative filtering algorithm can provide better online course recommendation for piano education. It plays a vital role in promoting the development of piano education and has strong practical significance in art and culture dissemination [6].

The research structure is as follows.  Section 1 is the introduction. It introduces the research background and research contribution.  Section 2 is the recent related work. It introduces the research on the recommendation algorithm.  Section 3 is the research method, which introduces the algorithm designed in detail.  Section 4 is the research results. The experimental data are used to test the performance of the proposed algorithm.  Section 5 is the conclusion, which summarizes the research and points out the future research direction.

## 2. Recent Related Work

The recommendation system has been widely concerned by scholars since its proposal. Batmaz et al. (2019) [7] showed that multiple previous recommendation systems rely on users' opinions and user item scores to realize the recommendation through collaborative filtering and content-based hybrid methods. Now, the relevant recommendations based on deep learning have been relatively perfect in theory and social perspective. Kiran et al. (2020) [8] showed that the previous recommendation system is no longer sufficient to meet the current requirements under social diversity.

At present, deep learning is developing rapidly. To better improve the performance of the recommendation system, a recommendation system combined with deep learning is undergoing a great change. For example, Ram et al. [9] introduced a video recommendation algorithm based on deep neural network. In journalism, scholars have introduced a wide and deep recommendation algorithm model. They show incomparable advantages over previous recommendation algorithms in social practice. Sivaramakrishnan et al. [10] found that most traditional recommendation algorithms have the disadvantage of data sparsity, which affects the recommendation performance.

Some results have been achieved in the previous recommendation methods. However, the recommendation accuracy needs to be improved with the development of the times. It is also the research necessity.

## 3. Materials and Methods

### 3.1. Types of Curriculum Resources

**Figure 1:**
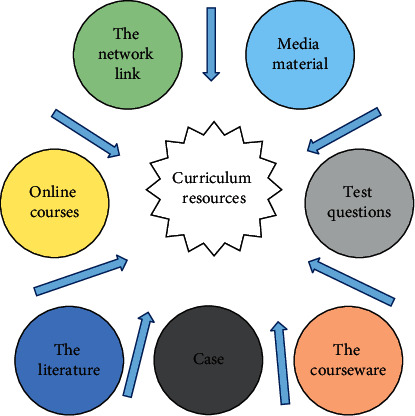
Types of curriculum resources.

There must be relevant course resources in the process of online course education. The so-called curriculum resources provide useable material conditions for teaching activities to make them more effective. Its species are also very rich.  Figure 1 displays several common types of curriculum resources [11].

Generally, media material is the smallest material unit for disseminating teaching information and the smallest element in constructing a teaching resource system. It generally covers various digital forms such as text, graphics, audio, and video. The test questions are a collection of test questions summarized according to the relevant evaluation criteria and conform to the educational theory. It mainly includes categories and types of questions, examination criteria, and relevant answers. The test paper is adopted to test students' learning knowledge. The types of courseware can be divided into single machine running and network-connected courseware. Its production process is categorized into learners, teaching objectives, and teaching content. Case is to integrate relevant media materials and teach with representative events. Literature is the relatively authoritative article published. A network course is to use a network for course teaching. Network link is to link the relevant knowledge points of various disciplines with the network address. Students can achieve the purpose of learning by accessing the network address. There are massive kinds of curriculum resources. No matter what form of teaching curriculum resources is adopted, the ultimate purpose is to provide better teaching services and enable students to learn efficiently. The rapid development of information networks enables students to summarize the process of online learning. For the method of finding learning course resources suitable for their own state, the recommendation algorithms used at this stage are introduced and analyzed [12].

### 3.2. Course Recommendation Algorithm

**Figure 2:**
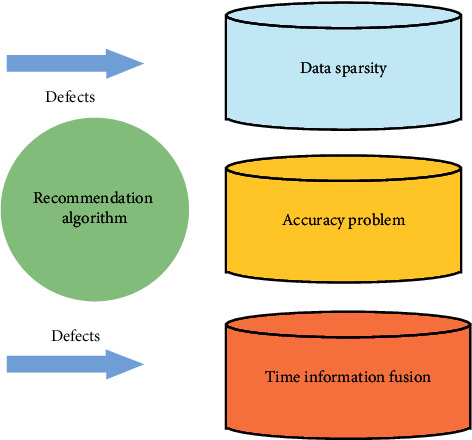
Problems in the course recommendation algorithm.

The present course recommendation algorithm has some problems due to its short development time.  Figure 2 displays the specific problem [13].

Specifically, the problem of data sparsity exists [14] because previous collaborative filtering algorithms analyze users' past information and make relevant recommendations on this basis. However, in the real use environment, users' scoring information on relevant items is incomplete, and they may not know whether the scoring is accurate or not. The scoring may become redundant items after the user completes the course, so the scoring operation will not be carried out, and there will be fewer data sources. There may be brushing points in online learning in real life because there is no way to conduct face-to-face teaching. Many factors lead to the problem of data sparsity. Learning resources are constantly updated and developed during learning, which is the reason for the accuracy problem of the recommendation algorithm. The learning state of users is also a dynamic process. The accuracy of the recommendation algorithm is not guaranteed under the joint action of the two [15]. Besides, time information differs significantly from other auxiliary information such as user information and course information. It has no interval, but it is increasing. Time information increases with the influx of other data, but it will not be input directly [16].

Regarding the above three problems, it is learned that the neural network can find high-level features on the way to learn the nonlinear network of users and goods, and the problem of data loss can be alleviated [17]. The interference of previous courses on new recommendations can be reduced by adding time elements to the neural collaborative filtering model, and the recommendation accuracy can be improved. In the face of the particularity of time information, other aspects need to be optimized to upgrade and improve the model. The following is a comprehensive analysis of the specific process content of recommended shortcomings [18].

### 3.3. Neural Network in Deep Learning

The integration of deep learning and online course recommendation has great practical value. Present training institutions have adopted online course learning. With its convenient and fast characteristics [19], teachers can still impart knowledge even at home, and people can learn tremendous knowledge without leaving home. Due to the lack of face-to-face communication between teachers and students, the knowledge dissemination can only take into account the learning status of the vast majority of people [20], and the matching degree between the courses taught and students may be gradually disconnected, making it difficult for students to learn the knowledge they want to learn from online courses. In contrast, course recommendations can better solve this problem. Students' personalized needs can be better understood by integrating deep learning and traditional recommendation algorithms [21].

**Figure 3:**
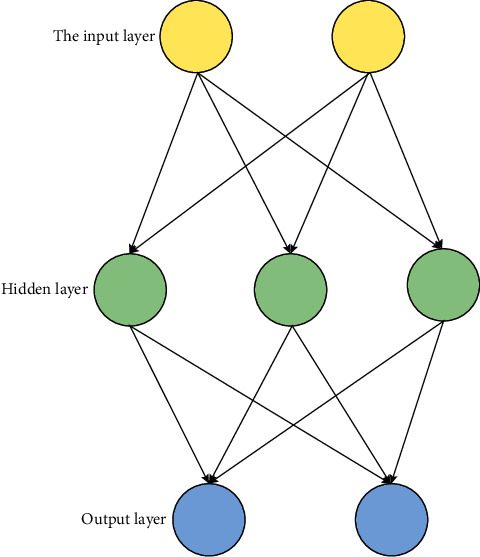
Structure of neurons.

The neural network is an algorithm model characterized by the ability to analyze complex problems and continuously adjust the relationship among its related nodes according to the characteristics of the problem to deal with complex issues well. Neurons are the most basic constituent units. Each neuron can have multiple inputs [22], and the effect of input data on the whole neuron can be adjusted by weight. The impact will become larger with the weight increase [23]. When the weight is negative, it needs to be continuously adjusted to make the output value close to the expected output value [24] to achieve the expected effect. In continuously adjusting the optimal weight, that is, the process of neural network learning and training, neurons can express both image format and text format. Among massive neurons, they have different responsibility classification forms, mainly input and output units and hidden units.  Figure 3 is a neural network composed of neurons.

In the neural network structure, the function of the input layer is mainly to receive external incoming signals, and the hidden layer is often between the input and output layers. Here, continuous learning and training are conducted through data processing and analysis. Finally, a suitable solution is found and output at the output layer. The self-regulation of weights according to the situation is the so-called autonomous learning ability [25].

The network structure determines the final result of the neural network. For example, there are some noteworthy points such as activation function, weight, and connection mode. Among the three, the activation function plays a crucial role in the learning and training of the artificial neural network. The following is mainly to introduce the activation function in the neural network structure. In the relevant network, the nodes of each layer convert the input data into output [26] and then go to the next node. Relevant activation functions are required to achieve good conversion between the last output and the input of the next layer. It is not difficult to find that the so-called activation function is actually the functional relationship between the two. The activation function is adopted to map the input of neurons to the output. In its application, its nonlinearity can change the input-output relationship of the network layer from linear to nonlinear, to explore a deeper relationship [27]. Sigmoid function, Tanh function, and Rectified Linear Unit (ReLU) function are commonly used activation functions. They are introduced in detail to pave the way for the later model design [28].

The value range of the Sigmoid function is between 0 and 1. The results obtained by the input function are expressed in the range between 0 and 1. The effect will be better when the difference of object characteristics is not particularly obvious. There is often the problem of gradient disappearance because of this function's large amount of calculation. The conversion between function S and input *x* is calculated as follows:

(1) $$\begin{array}{c} S\left(x\right)=\frac{1}{1+e^{-x}},\\ S^{\prime }=\frac{e^{-x}}{{\left(1+e^{-x}\right)}^{2}}=S\left(x\right)\left(1-S\left(x\right)\right). \end{array}$$

The Tanh activation function is to solve the disadvantage that the Sigmoid function is not symmetrical to the origin point [29]. It is still a hyperbolic function. In addition, it will have the disadvantage of gradient saturation. The conversion calculation equation between function Tanh and input *x* is as follows:

(2) $$\begin{array}{c} \text{Tan}h\left(x\right)=\frac{e^{x}-e^{-x}}{e^{x}+e^{-x}}. \end{array}$$

Compared with the Tanh and Sigmoid functions mentioned above, the value of the ReLU activation function will not be saturated, and the disadvantage of gradient descent will no longer exist. During calculation, the function is only adopted to find the maximum value. Besides, the index is not calculated to increase the probability of some other problems when the gradient is too large during learning and training, such as neuron inactivation. The conversion between function ReLU and input *x* is calculated as follows:

(3) $$\begin{array}{c} f\left(x\right)=\max\left(0,x\right),\\ \text{ReLU}\left(x\right)=\left\{\begin{array}{cc} x, & \text{if }x>0,\\ 0, & \text{if }x\leq 0. \end{array}\right. \end{array}$$

### 3.4. Collaborative Filtering Model Based on Neural Network

**Figure 4:**
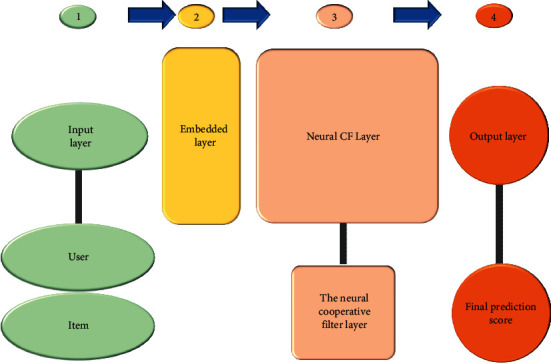
Neural collaborative filtering framework.

Collaborative filtering is a recommendation algorithm with good effect and frequent use [30]. Its working principle is to judge users' preferences by analyzing users' previous behavior data information and then use these preferences to classify users. Users with basically consistent preferences recommend similar items. The optimization of recommendations based on collaborative filtering has been optimized by researchers, hoping to improve the performance of the recommendation algorithm. Meanwhile, the research on collaborative filtering algorithm is also attracting much attention. Multiple MF methods are employed in the previous collaborative filtering algorithms to construct the hidden feature relationship between users and items. However, using a simple inner product cannot estimate the complex correlation between users and items, and the recommendation accuracy is affected. Based on this deficiency, the neural collaborative filtering model will be used. The neural network replaces the inner product operation in the previous MF and can well find the linear and nonlinear relationship between users and items.  Figure 4 displays the specific framework of the neural collaborative filtering algorithm.

Figure 4 suggests that the neural collaborative filtering framework has four levels. They are the input layer, embedded layer, neural cooperative filter layer, and finally output layer. They have different responsibilities. The input layer is responsible for inputting users and items, and it also needs to convert each user and item into a vector. For example, if there are *n* users, it will be converted into a vector of 1 × *n* users to convert it into a sparse vector. It is essential to multiply the input vector and the embedding matrix *p* after the input reaches the embedded layer. If there are *n* users, the embedded dimension is *m*. At this time, the embedded matrix size is *m* × *n*, which line refers to the embedding vector of the relevant user. After the obtained user embedding matrix and item embedding matrix are passed through the neural cooperative filter layer, the output layer is adopted for the final output of the results.

#### 3.4.1. Generalized MF Model (GMF)

MF essentially decomposes a matrix into the product of 1 or *n* matrices. It can solve the disadvantage of data sparsity in the previous collaborative filtering algorithms based on close neighbors. On the left of the neural collaborative filtering model is GMF. Compared with the previous MF, the GMF model uses the product of vectors, and the final result is also a vector. The equation of the GMF model is as follows:

(4) $$\begin{array}{c} p_{u}=\left[p^{1},\ldots \ldots ,p^{k}\right],\\ q_{i}=\left[q^{1},\ldots \ldots ,q^{k}\right],\\ \varphi \left(p_{u},q_{i}\right)=p_{u}\cdot q_{i}\\ =\left[p^{q}q^{q},p^{2}q^{2},\ldots \ldots ,p^{k}q^{k}\right],\\ y_{ui}=\alpha_{\text{out}}\left(h\left(p_{u}\cdot q_{i}\right)\right), \end{array}$$

where *p*_*u*_ and *q*_*i*_ are input to the embedded layer to obtain the potential feature vectors of users and items. The correlation between users and items is obtained after the inner product, and then, the final prediction result is obtained through the output layer. *α*_out_ here is the activation function of Sigmoid; *h*() generally represents the output layer.

#### 3.4.2. Artificial Neural Network with the Forward Structure

Multilayer perceptron (MLP) is a frequently used artificial neural network, with a forward structure. It consists of multiple layers, including the input, hidden, and output layers. Layers are connected by full connection, and forward propagation is used for data transmission between layers. This algorithm is adopted to calculate the output of each, and then, the back propagation algorithm is employed to find the best parameters. If the data information input in the input layer is (*a*, *b*), the weight of the hidden layer is *w*_1_, and the weight of the output layer is *w*_2_, the calculation equation of the *α*_*h*_ and *α*_out_ functions obtained from the hidden layer is

(5) $$\begin{array}{c} \alpha_{h}=f_{1}\left(w_{1}\cdot \alpha \right),\\ \alpha_{\text{out}}=f_{2}\left(w_{2}\cdot \alpha_{h}\right). \end{array}$$

**Figure 5:**
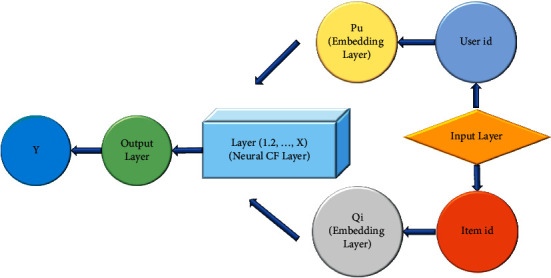
MLP framework.

Next to the neural collaborative filtering model is the MLP framework. The more profound correlation between users and items can be found through the unique network structure of the MLP framework.  Figure 5 presents its frame diagram.

Figure 5 reveals that MLP starts from the two points of user and item, finds the correlation between them, and takes the correlation as the final output data to optimize the collaborative filtering model. The framework shows that it inputs the feature vectors Pu and Qi of users and items into the multilayer neural network to find the final score and demerit. There is a certain difference between it and the above GMF model in processing user and item embedding vectors in the embedded layer.

### 3.5. Construction of Neural Collaborative Filtering Model Integrating Time Auxiliary Information

In practice, compared with other resources, the variable of time plays a more significant role in online course resources. With the change of time, the time information element is often not taken into account when the previous recommendation algorithms are used. Generally, the default is that the user's preference is fixed and will not change. In the real teaching process, it is known that users' preferences will change with the change of time, so only integrating deep learning is still not enough. Adding relevant auxiliary information can make the whole recommendation effect better. Time information is integrated into the recommendation algorithm to better explore users' dynamic preferences. Compared with other online course resource recommendation models, the most significant difference of the designed online resource recommendation model is time impact. With the continuous advancement of the learning stage and the dynamic updating of course resources, the learning directions that each user has to face at different stages are very different. The courses appear far away and the viewing frequency is not high, so the recommendability will not be very high, which will be presented in this model. The time information is classified by *K*-means clustering algorithm and combined into the MLP model and GMF model as time feature vector to design a neural collaborative filtering model integrating time auxiliary information. It can provide users with more accurate dynamic recommendations and optimize their results.

In the K-means clustering algorithm process, first, several numbers are randomly selected on the dataset as the initial central value. Then, the numbers in the dataset are compared with the obtained central value. The way of comparison is to calculate the distance between the center value and each number. The calculation results record the correlation between the number and the center and assign each number to the nearest cluster center. Each number next to the cluster center represents a cluster, and then, a number is allocated. The algorithm will be repeated all the time when the cluster center changes. The algorithm is over when the central value of clustering is not changed. The data can be categorized into several categories through this method. The distance *y* is expressed by the equation:

(6) $$\begin{array}{c} y=\min\sum_{i=1}^{n}\underset{j=1,2,..k}{\min}{\left\|x_{i}-\mu \right\|}^{2}. \end{array}$$

**Figure 6:**
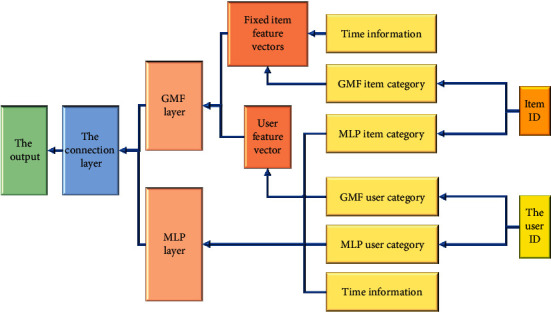
Improved Neu MF model framework.

Here, *xi* algorithm is the data in the dataset, *μ* is the cluster center value, and K is the number of initial clusters. *K*-means clustering algorithm can find the data correlation in messy data information for classification. Time is a piece of special auxiliary information that can become larger without limitation, which leads to the unilateral use of time as auxiliary information in the model, and the complexity of the model will be greatly improved. At this time, the problem of introducing a *K*-means clustering algorithm can be well solved. *K*-means is adopted to map the time information to an interval and input it as auxiliary information. The basic framework of the designed Improved Neu Matrix Factorization (Improved Neu MF) model has been formed.  Figure 6 is the model framework.

During the learning and training of the above model, the convergence speed of training may be affected and slow down with the increase in the number of network layers. Batch standardization can use the means of relevant specified standards to act the distribution of input values of neural networks at each layer on appropriate standards, to speed up the whole training speed and reference the batch standardization layer into the MLP model. The problem of overfitting can also be alleviated when the training speed is improved. After linear learning and nonlinear learning, the deeper feature vectors are connected and output by the Sigmoid function. The relevant equation of Improved Neu MF is as follows:

(7) $$\begin{array}{c} X^{\text{GMF}}=p_{u}^{\text{GMF}}\cdot q_{i}^{\text{GMF}}+p_{i}^{\text{GMF}}\cdot t_{ui}^{\text{GMF}},\\ X^{\text{MLP}}=\alpha_{L}\left(W_{L}^{T}\left(\alpha_{L-1}\left(\cdots \alpha_{2}\left(W_{2}^{T}\left[\begin{array}{c} p_{u}^{M}\\ q_{i}^{M}\\ t_{ui}^{M} \end{array}\right]+b_{2}\right)\cdots \right)\right)+b_{L}\right),\\ y_{ui}=\sigma \left(h^{T}\left[\begin{array}{c} X^{\text{GMF}}\\ X^{\text{MLP}} \end{array}\right]\right),\\ L=\frac{1}{n}\sum_{i=1}^{n}{\left|y_{ui}-y_{\text{real}}\right|}^{2},\\ L=\sum_{\left(u,i\right)\in D\cup D^{-}}\left[y_{ui}\log\;y_{\text{real}}-\left(1-y_{ui}\right)\log\left(1-y_{\text{real}}\right)\right], \end{array}$$

where *p*_*u*_^*M*^ and *p*_*u*_^GMF^ are the input representations of users in MLP and GMF, respectively; *t*_*ui*_^GMF^ and *t*_*ui*_^*M*^ are input using the viewing time classification of *u* and items *i* in GMF and the viewing time classification information in MLP as one-dimensional vectors, respectively.

### 3.6. Simulation Experiment

At present, there are multiple online course platforms, such as Immoc, Cloud Classroom of NetEase, and so on. The Scrapy framework in python is adopted to crawl the data on Immoc, and a total of 289333 pieces of data are obtained. Among these data, the course records of randomly selected users are more than 13. These data are obtained on the premise of protecting users' privacy, which are used as the production of training set. After that, 6689 users and all 478 courses in the dataset are obtained as the dataset. The root mean square error (RMSE) and mean absolute error (MAE) of collaborative filtering (CF) algorithm, Neu Matrix Factorization (NeuMF), and Improved Neu MF are compared, and their desirability is analyzed from the data.

## 4. Results

### 4.1. Comparison Results of RMSE and MAE

There are 6689 users and all 478 courses in the dataset. Any course seen by each user is selected from as the test set and others are taken as the training set. Hundred courses not seen by users are selected and added to the test set and tested by NeuMF, Improved Neu MF, and CF.  Table 1 presents the obtained RMSE and MAE.

**Figure 7:**
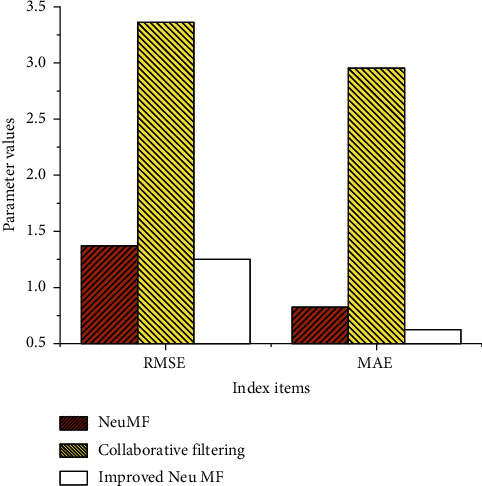
Comparison of RMSE and MAE gap.

Table 1 shows that the RMSE and the MAE of the Improved Neu MF are 1.251 and 0.625, respectively. Compared with the other two algorithms, the error is smaller and its budget accuracy is higher. They are compared in the dotted line diagram to better highlight the gap between them, as shown in Figure 7.

Figure 7 shows that the RMSE and MAE of the Improved Neu MF algorithm model are about 1 lower than Neu MF and CF, which has obvious advantages.  Table 1 suggests that the model accuracy of the algorithm is excellent.

### 4.2. Experimental Comparison between Normalized Discounted Cumulative Gain (NDCG) and Hits Ratio (HR)

HR can better reflect the recommendation accuracy, and NDCG can better reflect the order of recommended items. The three algorithms are compared experimentally.  Table 2 suggests the results.

**Figure 8:**
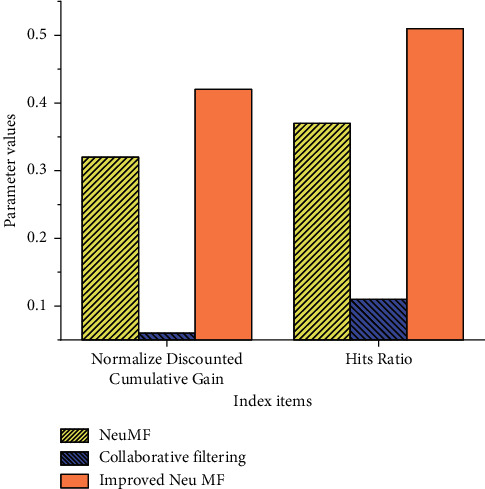
Comparison of HDCG and HR.

Table 2 shows that the NDCG of the designed Improved Neu MF reaches 0.42 and the HR reaches 0.51. Compared with the two other algorithms, the NDCG of collaborative filtering is 0.06, and the HR is 0.11; the NDCG of Neu MF is 0.32, and the HR is 0.37. The designed algorithm is leading in both evaluation indexes, which explains the addition of time information. The designed algorithm can find more suitable courses and recommend them to users. With the above experimental method, their gap amplitude is plotted to observe the performance improvement amplitude more clearly, as shown in Figure 8.

Gap amplitude comparison shown in Figure 8 shows that the NDCG and HR of the designed Improved Neu MF algorithm model are higher than the compared algorithm. According to Table 2, HR is 0.4 higher than the lowest collaborative filtering, and NDCG is 0.1 higher than the lowest collaborative filtering. It shows that with the addition of time information, the performance of the designed algorithm model is better.

## 5. Conclusion

Under the background of sustainable economic development, the demand for art and culture at all levels in multiple countries is also gradually showing a straight-line upward trend. The piano is a crucial member of music. There are many teaching problems in piano online education courses.

The problem of data sparsity is further solved on the premise of collaborative filtering. The advantages of deep learning and traditional collaborative filtering recommendation algorithm are combined to design a neural collaborative filtering algorithm. The traditional MF is employed to find the linear relationship between users and items. The multilayer neural network is adopted to find the nonlinear relationship between users and items to reduce the interference of missing data values and optimize the accuracy. Curriculum resources and learning should be changed accordingly during teaching. The K-means clustering algorithm applies time data to neural collaborative filtering algorithms to optimize online course resources to the greatest extent. The relevant evaluation indexes are selected after the experiment. Simulation verification is carried out on the relevant datasets. The test results show that the model has an optimized performance. Based on deep learning, the model designed by using a neural collaborative filtering algorithm can provide better online course recommendations in piano education. It plays a crucial role in promoting the development of piano education and has strong practical significance for disseminating art and culture. However, in selecting experimental data, only individual online learning platform data are selected, which may exert a certain impact on the experimental results. The sample data source can be increased in the follow-up exploration to make the experimental results more convincing.

## Figures and Tables

**Table 1:** Comparison results of RMSE and MAE.

Use item	NeuMF	Collaborative filtering	Improved Neu MF
RMSE	1.372	3.362	1.251
MAE	0.825	2.953	0.625

**Table 2:** Experimental results of HDCG and HR.

Use item	Normalize discounted cumulative gain	Hits ratio
NeuMF	0.32	0.37
Collaborative filtering	0.06	0.11
Improved Neu MF	0.42	0.51

## Data Availability

The data used to support the findings of this study are included within the article.
